# A Co-Doped Carbon Dot/Silver Nanoparticle Nanocomposite-Based Fluorescence Sensor for Metformin Hydrochloride Detection

**DOI:** 10.3390/nano12081297

**Published:** 2022-04-11

**Authors:** Thi-Hoa Le, Ji-Hyeon Kim, Sang-Joon Park

**Affiliations:** Department of Chemical and Biological Engineering, Gachon University, Seongnam 13120, Korea; lehoa290792@gachon.ac.kr (T.-H.L.); jihyeon@gachon.ac.kr (J.-H.K.)

**Keywords:** inner filter effect, fluorescence recovery, NPCD/AgNP nanocomposites, metformin hydrochloride

## Abstract

In this study, a fluorescence sensor based on nitrogen and phosphorus co-doped carbon dot/silver nanoparticle (NPCD/AgNP) nanocomposites was developed for metformin hydrochloride (MFH) detection. We first utilized the reducing nature of the NPCDs to prepare AgNPs from Ag^+^ and subsequently prepare NPCD/AgNP nanocomposites. The nanocomposite material was characterized by various methods, including electron microscopic methods (SEM and TEM), spectroscopic methods (UV-Vis, PL, FTIR, and XPS spectroscopy), light scattering (ELS), and XRD. Further, we utilized the enhanced fluorescence of the NPCDs as well as the overlap between the fluorescence emission spectrum of the NPCDs and the absorption spectrum of the AgNPs to use the NPCD/AgNP nanocomposites as an effective inner filter effect (IFE) pair for sensing MFH. The IFE between NPCDs and AgNPs in the nanocomposite material resulted in a significant quenching of the fluorescence intensity of the nanocomposites compared to that of the pure NPCDs. However, the fluorescence was recovered when MFH was introduced into the nanocomposite solution. The fluorescence intensity of the nanocomposites increased linearly as the MFH concentration increased from 2 to 100 µg/L. This detection method showed good sensitivity compared to other methods. It also showed high selectivity and high sensing potential for MFH in human serum and yielded acceptable results.

## 1. Introduction

Diabetes is a chronic health condition that affects the body’s metabolism. Most of the food that is consumed is broken down into glucose and released into the bloodstream. Increased blood sugar levels signal the pancreas to release insulin, which primarily governs the cellular uptake of blood sugar in the body for its use as energy. The body of a diabetic person either cannot effectively use the produced insulin (type I diabetes) or cannot produce enough insulin (type II diabetes) [1,2,3]. When the insulin is insufficient or when cells stop responding to insulin, blood sugar levels increase. Over time, this can cause serious health problems. The number of diabetic patients is increasing every year and is predicted to reach 700 million by 2045 [4].

Metformin hydrochloride (MFH) is an effective antidiabetic drug from the biguanide class and has been used to control blood glucose levels in patients with type II diabetes [5,6]. MFH inhibits complex I (NADPH: ubiquinone oxidoreductase) formation in the mitochondrial respiratory chain, thereby increasing the ratio of cellular AMP to ATP. This in turn activates the AMP-activated protein kinase (AMPK) and regulates the AMPK-mediated transcription of the target genes. This eventually prevents hepatic gluconeogenesis and enhances insulin sensitivity and fatty acid oxidation, ultimately leading to a decrease in glucose levels. Therefore, MFH is the first choice for diabetic patients according to international instructions [7]. In addition, the anti-aging and anti-cancer effects of MFH have attracted considerable attention from scientists [8]. However, excess MFH in the body is excreted through urine, while the accumulation of MFH in the human body can cause side effects [9]. Hence, it is necessary to develop effective methods for monitoring MFH elimination in diabetic patients.

Many methods, such as spectrophotometric [6,10,11], voltammetric [12], chromatographic (e.g., HPLC) [13], and electrochemical [14] methods, have been reported for the detection and quantification of MFH. Among these techniques, the specificity, sensitivity, operational simplicity, and ease of sample preparation render fluorescence-based techniques the most promising and attractive for sensing different analytes. In this study, we developed a fluorescence sensor for the detection of MFH by harnessing the inner filter effect (IFE) between nitrogen and phosphorus co-doped carbon dots (NPCDs) and silver nanoparticles (AgNPs).

An overlap between the absorption spectrum of an acceptor and the fluorescence excitation or emission spectrum of a donor can result in an IFE, wherein the fluorescence intensity of the donor is quenched [15]. IFEs can be further classified into primary and secondary IFEs. In the former, the excitation beam is attenuated by the volume of the sample, while in the latter the emission of the fluorophore is attenuated by a molecule present in the solution. Usually, the IFE is an undesirable phenomenon in fluorescence measurements [16]. However, in this study, we utilized the IFE to design a “turn on” fluorescence-based sensor, which could pave the way for developing new and effective strategies for sensing analytes. Compared to “turn off” fluorescence sensors, the “turn on” fluorescence sensors are more preferable because the likelihood of false positives is reduced.

Previous studies have shown that the unique physical and chemical properties of carbon dots (CDs) and silver nanoparticles (AgNPs) render them an outstanding pair for exhibiting IFE [17,18,19]. CDs have distinct properties, including outstanding photostability and photoluminescence, tunable surface functionalities, high solubility, low toxicity, and good biological properties, such as biocompatibility and cell membrane permeability; thus, they can serve as excellent electron donors and acceptors [20,21,22].

AgNPs have attracted considerable attention owing to their unique optical, thermal, electrical, and biological properties. Particularly, AgNPs have a tunable absorption spectrum and extremely high extinction coefficient [23], rendering them an appropriate acceptor for achieving effective IFE with CDs.

Recently, carbon/AgNP-based nanomaterials have been employed for various purposes; for instance, graphene oxide/AgNP nanocomposites have been used as antibacterial agents [24], graphene oxide/AgNP hybrids act as effective substrates for surface-enhanced Raman spectroscopy [25,26], and mesoporous carbon/AgNP nanohybrids have been employed as nonenzymatic hydrogen peroxide sensors [27]. However, the synthesis of carbon/AgNP-based nanomaterials requires additional reductants such as sodium borohydride, sodium citrate, and ascorbic acid to synthesize AgNPs, and this is a time-consuming and complex process. Moreover, the reductant ions on the surface of AgNPs can limit their applications. 

In this study, we fabricated a fluorescence sensor based on the IFE in a CD/AgNP nanocomposite material for MFH detection. Most fluorescence sensors are based on the fluorescence of CDs. The abundant number of hydroxyl and carboxyl groups make the CDs good reducing and stabilizing agents for the synthesis of metal nanomaterials [28]. The negatively charged carboxyl groups on the CDs can stabilize the metal particles in solution [29], while the hydroxyl groups allow CDs to act as green reducing agents in the synthesis of metal nanoparticles [30]. Therefore, the fluorescence of CDs and their possible function as reducing and stabilizing agents should be harnessed in the design and fabrication of CD/AgNP nanocomposite-based sensors.

**Scheme 1:**
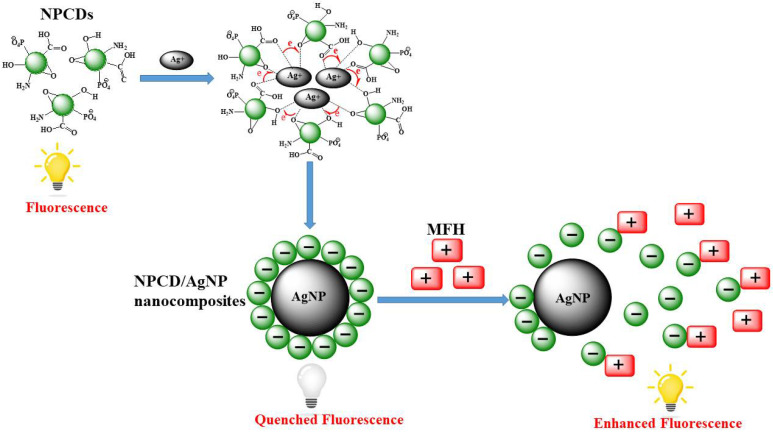
Illustration of the NPCD/AgNP-nanocomposite-based system used for MFH detection.

NPCDs were successfully synthesized in our previous study [20]. The fluorescence output of NPCDs was significantly enhanced compared to that of the pristine CDs. The numbers of negatively charged functional groups and amine groups increased due to the presence of nitrogen and phosphorus. The amine groups has a strong binding affinity toward the surface of the AgNPs; thus, NPCDs can bind and increase the negative surface charge of AgNPs and enhance the IFE between the NPCDs and these nanoparticles [31,32]. Therefore, in this study, we used NPCDs as fluorophores and reducing agents instead of pristine CDs. The mechanism of “turn on” fluorescence for MFH detection is illustrated in Scheme 1.

## 2. Chemicals and Experiments

### 2.1. Chemicals

All chemicals, namely (NH_4_)_2_HPO_4_, citric acid monohydrate, AgNO_3_, MFH, glycine, alanine, methionine, cysteine, lysine, glucose, saccharose, Na_2_SO_4_, KNO_3_, CaCl_2_, MgCl_2_, FeCl_2_, human serum, and deionized (DI) water, were purchased from Sigma-Aldrich (MA, USA). 

### 2.2. Instruments

Photoluminescence (PL) and UV–Vis spectra were obtained on a QuantaMaster TM 50 PTI (Photon Technology International, San Diego, CA, USA) spectrofluorometer and a G1103A (Agilent, Santa Clara, CA, USA) UV–Vis spectrophotometer. The structure and morphology of the samples were characterized by transmission electron microscopy (TEM; Tecnai, F30S-Twin, Hillsboro, OR, USA) and scanning electron microscopy (SEM; S-4700, Hitachi Ltd., Tokyo, Japan). The particle size distribution was determined by electrophoretic light scattering (ELS; Photal Otsuka Electronics, ELS 8000, Osaka, Japan). Fourier transform infrared (FTIR) spectroscopy was performed on a Nicolet 6700 spectrometer (Thermo Scientific, Waltham, MA, USA). X-ray photoelectron spectroscopy (XPS) and powder X-ray diffraction (XRD) were performed on an X-ray photoelectron spectrometer (PHI 5000, Chigaski, Kanagawa, Japan) and an X-ray source (Rigaku/Smartlab, Tokyo, Japan), respectively.

### 2.3. Preparation of NPCDs

NPCDs were fabricated in our previous paper [20]. Briefly, a mixture of (NH_4_)_2_HPO_4_ (5 g), citric acid monohydrate (2 g), and DI water (30 mL) was poured into a 50 mL Teflon-lined stainless-steel autoclave and heated at 180 °C for 4 h. After cooling, the solution was filtered through a 0.22 µm polyethersulfone membrane and then dialyzed using a dialysis bag (MWCO: 1000 Da) for 48 h. The dialyzed, light-violet-colored solution was lyophilized to obtain a powdery substance.

### 2.4. Preparation of NPCD/AgNP Nanocomposites

The NPCD solution (1.2 mL, 0.075 g/mL) was added to DI water (8.8 mL) and the mixture was sonicated for 30 min and heated to 90 °C. Following this, AgNO_3_ (200 µL, 0.15 M) was added to the solution and the mixture was stirred for 40 min. During stirring, the color of the solution first changed to light yellow and then to brown, indicating the successful formation of AgNPs. 

### 2.5. Fluorescence Sensing of MFH

The MFH sensing experiments were performed at room temperature. First, a solution of NPCD/AgNP nanocomposites (3 mL, 3.03×10^−3^ g/mL) was prepared. Then, different amounts of MFH solution were added dropwise into the NPCD/AgNP solution to obtain final concentrations of MFH from 2 to 100 µg/L. The volume was filled up to the mark using DI water, and the pH was controlled at 7.4. The samples were allowed to equilibrate for 10 min before performing fluorescence measurements (λ_ex_ = 330 nm). To determine the standard deviations, the experiment for each concentration was repeated three times.

### 2.6. Detection of MFH in Human Serum

First, human serum (30 µL) was added to DI water (1970 µL) to obtain a 2 mL human serum solution. Then, the NPCD/AgNP solution (1000 µL, 9.1 × 10^−3^ g/mL) was added to the human serum solution and the solution was sonicated for 30 min. A certain volume of the MFH solution was added dropwise into the solution, followed by dilution with DI water and a pH control of 7.4 before the fluorescence measurements. MFH in the human serum samples was detected using the method described above.

### 2.7. Selectivity

The fluorescence response from other amino acids, carbohydrates, and electrolytes was studied to investigate the specificity of the NPCD/AgNP nanocomposites-based fluorescence sensor for MFH detection.

## 3. Results and Discussion

### Characterization of NPCD/AgNP Nanocomposites

**Figure 1:**
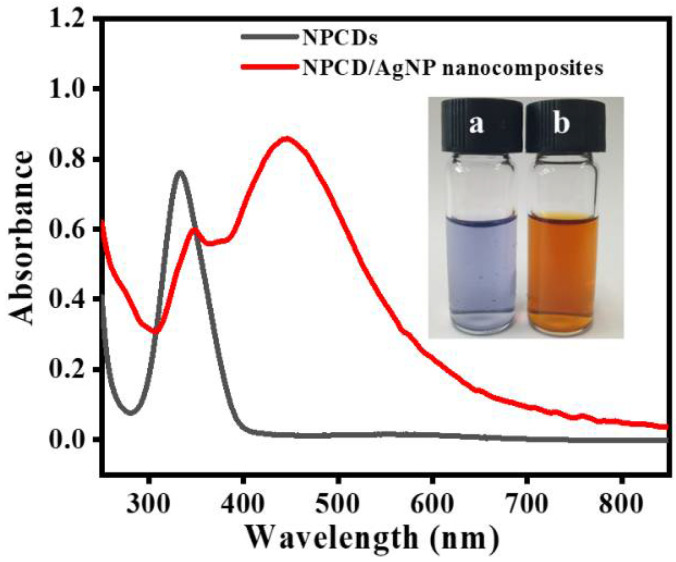
UV–Vis spectra of the aqueous solution of the NPCDs and NPCD/AgNP nanocomposites (inset: color of the aqueous solutions of (**a**) NPCDs and (**b**) NPCD/AgNP nanocomposites).

The optical properties of the NPCDs and NPCD/AgNP nanocomposites were examined by UV–Vis absorption and fluorescence spectroscopy. The light violet NPCD solution exhibited an absorption peak at 335 nm (Figure 1), which was the same as the peak position reported previously [33,34]. In the spectrum of the brown aqueous solution of the NPCD/AgNP nanocomposites, the NPCD peak was observed to shift to a longer wavelength (345 nm), along with the appearance of a new and prominent peak at 446 nm in the visible range. This was in good agreement with the surface plasmon resonance peak from 400 to 450 nm observed for AgNPs synthesized using different reducing agents, such as silver trisodium citrate and sodium borohydride [35]. This confirmed the successful reduction of NPCDs to form AgNPs.

**Figure 2:**
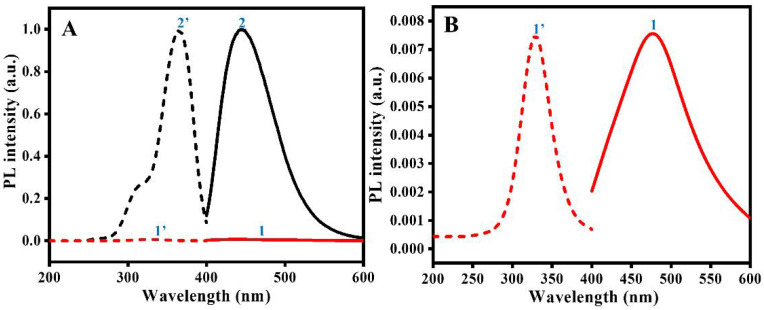
(**A**) Excitation fluorescence spectra (1′, 2′) and emission fluorescence spectra (1, 2) of the NPCDs (2′, 2) and NPCD/AgNP nanocomposites (1′, 1), (**B**) magnified excitation and emission fluorescence spectra of the NPCD/AgNP nanocomposites.

The fluorescence emission spectrum of the NPCD solution showed a peak at 445 nm upon the excitation peak at 360 nm (Figure 2). In the emission spectrum of NPCD/AgNP nanocomposites, this peak was red-shifted to 475 nm, with the excitation peak being visible at 330 nm. Moreover, the fluorescence intensity of the NPCD/AgNP nanocomposites was significantly quenched compared to that of the NPCDs. This quenching can be explained by the IFE in the nanocomposite material, which is clearly described in Section 2.1.

**Figure 3:**
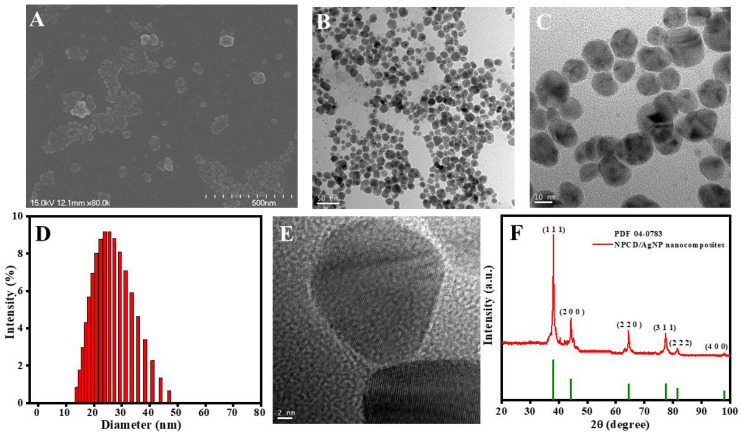
(**A**) SEM image, (**B**,**C**) TEM images, and (**D**) ELS analysis of the NPCD/AgNP nanocomposites. (**E**) High-resolution TEM image and (**F**) powder XRD pattern of NPCD/AgNP nanocomposites.

SEM, TEM, and ELS were used to analyze the morphology and size of the NPCD/AgNP nanocomposites. The synthesized AgNPs were spherical and relatively monodispersed, and the size distribution ranged from 14 to 47 nm, with an average size of 25.9 nm (Figure 3A–D). The crystal structure of the AgNPs shown in a high-resolution TEM image (Figure 3E) was further characterized via powder XRD.

Figure 3F shows the powder XRD patterns of the NPCD/AgNP nanocomposites. The six peaks at 38.09°, 44.27°, 64.48°, 77.31°, 81.55°, and 98.01° can be assigned the (111), (200), (220), (311), (222), and (400) planes, with lattice fringe distances of 2.36, 2.04, 1.44, 1.23, 1.17, and 1.02 Å, respectively. Therefore, there is a high degree of similarity between the XRD pattern of the synthesized nanocomposites and that of pure crystalline Ag (0) with a face-centered cubic crystal structure (PDF 04-0783).

**Figure 4:**
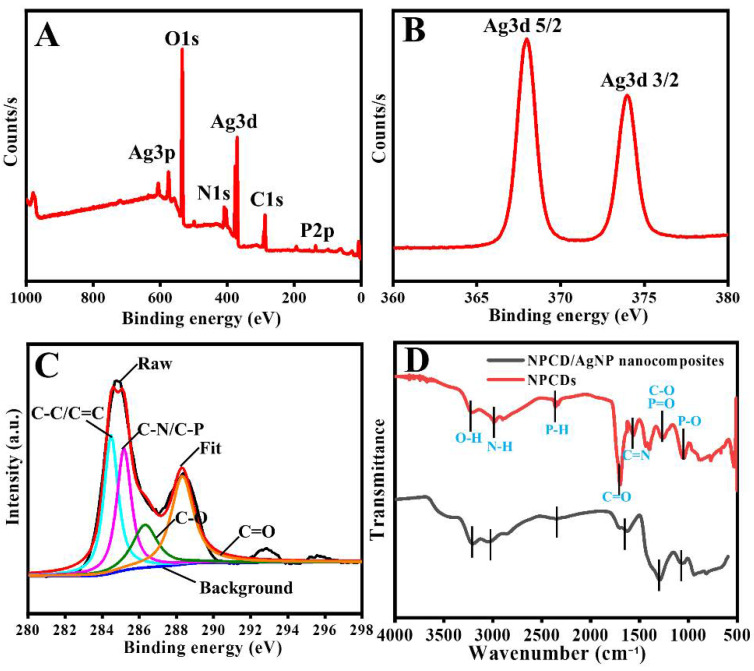
(**A**–**C**) XPS spectra of NPCD/AgNP nanocomposites. (**D**) FTIR spectra of the NPCDs and the NPCD/AgNP nanocomposites.

The XPS profile of the nanocomposites shows typical peaks corresponding to Ag 3p (Ag 3p1/2, and Ag 3p3/2), O 1s, N 1s, Ag 3d, C 1s, and P 2p at 604, 573, 533, 402, 369, 285, and 131 eV, respectively (Figure 4A). The weight percentages of Ag, C, O, N, and P were determined to be 9.66%, 39.65%, 37.58%, 8.82%, and 4.29%, respectively. The high-resolution XPS profiles of the Ag 3d and C 1s peak are shown in Figure 4B,C. The Ag 3d_5/2_ and Ag 3d_3/2_ peaks at 367 and 374 eV, respectively, correspond to the metallic state of Ag (0). Because of the C = O, C–O, C–N/C–P, and C–C/C = C, which have a sp^2^ graphitic structure [36], the C1s peak can be deconvoluted into four peaks at 288.3, 286.3, 285.2, and 284.5 eV, respectively. The high-resolution XPS profiles of P 2p and N 1s, which were similar to those in our previous study [20], further confirm the existence of these bonds on the surface of the NPCDs covering the AgNPs. The FTIR spectra of the NPCDs and the NPCD/AgNP nanocomposites (Figure 4D) further support these assignments. The peaks at 3300–2500, 3000–2800, 2332, 1702, 1600, 1259, 1211, and 1047 cm^−1^ in the FTIR spectrum of the NPCDs correspond to the stretching vibrations of O–H, N–H, P–H, C = O, C = N, P = O, C–O, and P–O bonds, respectively, belonging to the carboxyl, hydroxyl, amine, and phosphate group. These peaks can also be observed in the spectrum of the NPCD/AgNP nanocomposites, which indicates that after behaving as a reducing agent in the synthesis of AgNPs, the NPCDs could act as one of the components to form a nanocomposite material with AgNPs. However, there are some differences in the range and sharpness of some peaks in the spectrum of the nanocomposites compared to that of pure NPCDs. This shows that NPCDs contain abundant negative charged functional groups and amine groups, they bind and have interactions with the surfaces of AgNPs, and they increase the negative charge of AgNPs. These interactions did not change the chemical bonds in functional groups of NPCDs but can affect the bonding vibration frequency and lead to variations in the spectrum.

## 4. Detection of MFH

### 4.1. Mechanism of Sensing

**Figure 5:**
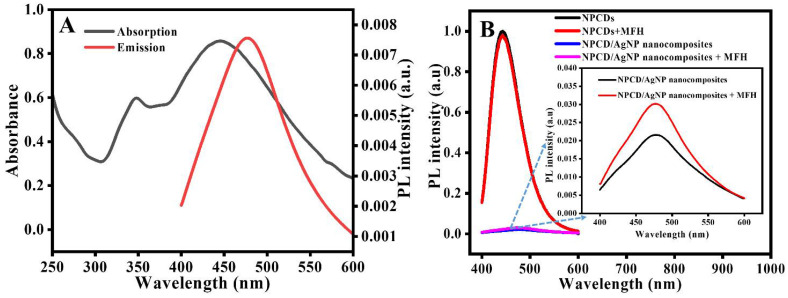
(**A**) Overlap of the absorption and emission spectra of the NPCD/AgNP nanocomposites. (**B**) Fluorescence response of the NPCDs and the NPCD/AgNP nanocomposites (inset: magnified fluorescence spectra of the NPCD/AgNP nanocomposites) in the presence and absence of MFH (15 µg/L).

**Figure 6:**
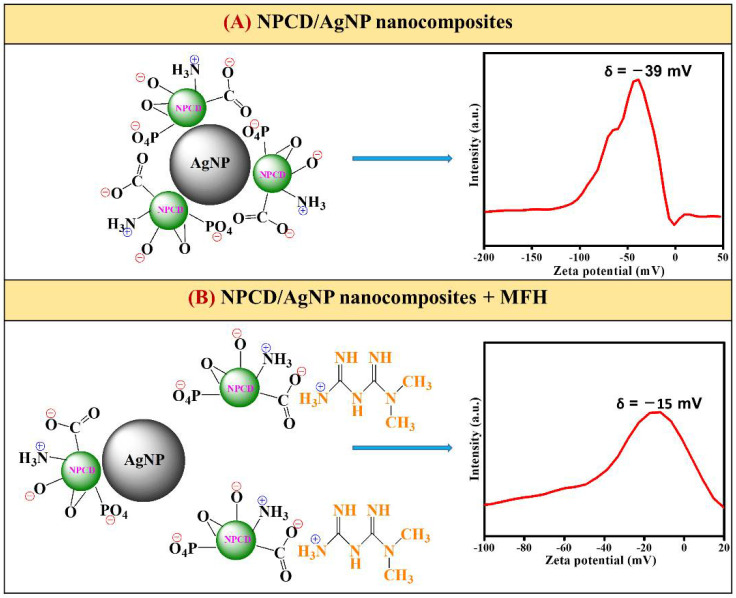
The basis of the zeta potential values of (**A**) the NPCD/AgNP nanocomposites and (**B**) the NPCD/AgNP nanocomposites + MFH (50 µg/L) solution.

Because of the presence of the AgNPs, the nanocomposites exhibited an absorption peak at 446 nm (Figure 1). The emission peak at 475 nm was observed because of the NPCDs (Figure 2). Therefore, there was an overlap between the absorption and emission peaks, as illustrated in Figure 5A, and this overlap triggered the IFE, leading to a dramatic quenching of the fluorescence intensity of the NPCD/AgNP nanocomposites compared to that of the pristine NPCDs. The introduction of MFH to the NPCD/AgNP nanocomposites “turned on” the fluorescence. Interestingly, this phenomenon was not observed for the pure NPCDs. In other words, the addition of MFH did not alter the fluorescence intensity of the NPCDs but “turned on” (recovered) the fluorescence of the NPCD/AgNP (Figure 5B). Scheme 1 and Figure 6 clearly explain this difference. The NPCDs have an overall negative charge because of the negatively charged function groups, such as the carboxyl, hydroxyl, and phosphate groups on its surface. In a previous study, we demonstrated that the NPCDs have a negative zeta potential [20]. Thus, besides acting as a reductant in the synthesis of the AgNPs, NPCDs also acted as one of the components in the NPCD/AgNP nanocomposites. The NPCDs existed around the surface of the AgNPs, rendering the nanocomposites negatively charged. Upon the addition of the positively charged MFH molecules [37], the zeta potential of the nanocomposites changed from −39 to −15 eV, suggesting the existence of electrostatic interactions between the nanocomposites and MFH molecules. Because of the electrostatic interactions, the NPCDs were separated from the surfaces of the AgNPs, weakening the IFE between them and recovering the fluorescence intensity of the NPCDs. This is the basic mechanism for the design and fabrication of a “turn on” fluorescence sensor for MFH detection.

**Figure 7:**
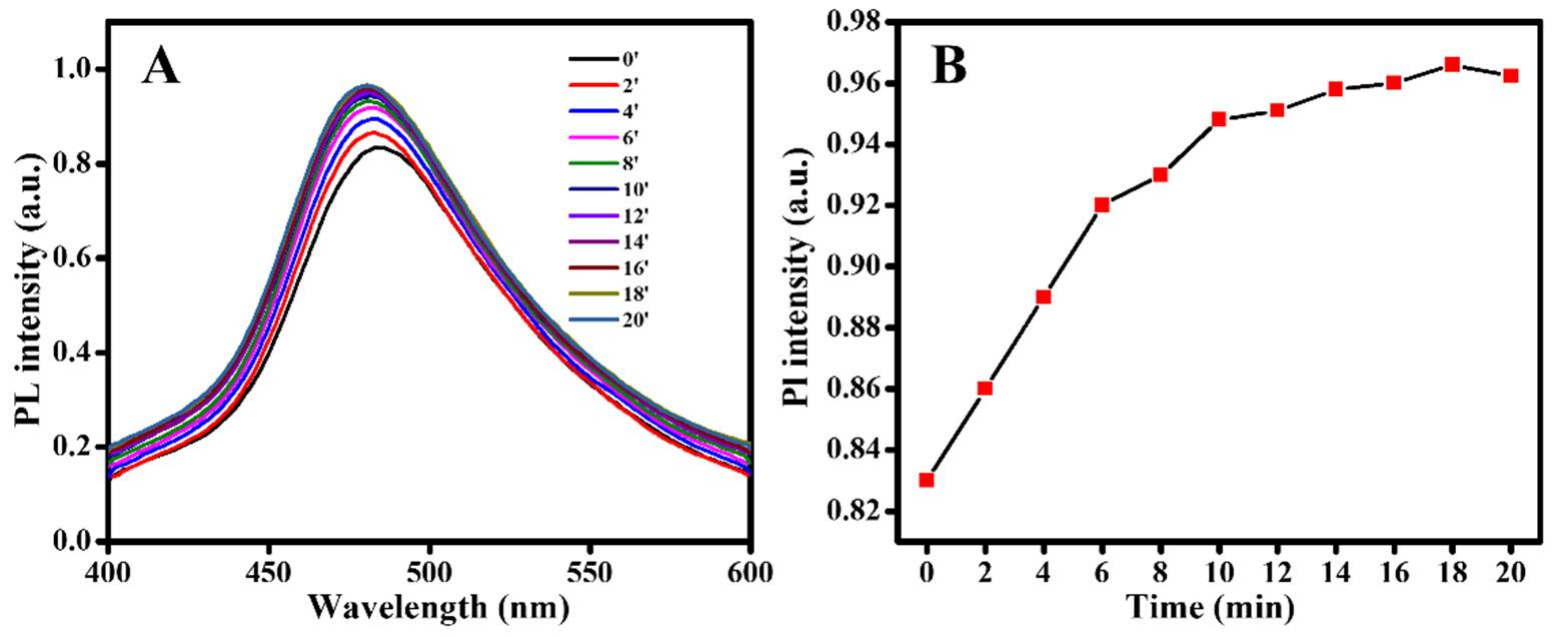
(**A**) Fluorescence spectra and (**B**) changes in the relative fluorescence intensity (I/I_0_) of the NPCD/AgNP nanocomposties in the presence of MFH (10 µg/L) as a function of time.

To study the kinetics of the IFE in the NPCD/AgNP nanocomposites, the fluorescence intensity of the nanocomposite solution was recorded every 2 min upon the addition of 10 µg/L MFH. A steady increase in the fluorescence intensity of the nanocomposites was observed from 2 to 10 min (Figure 7). Thereafter, the intensity did not change significantly and remained relatively stable. Therefore, 10 min was determined to be the optimal time for experiments.

### 4.2. Sensing

**Figure 8:**
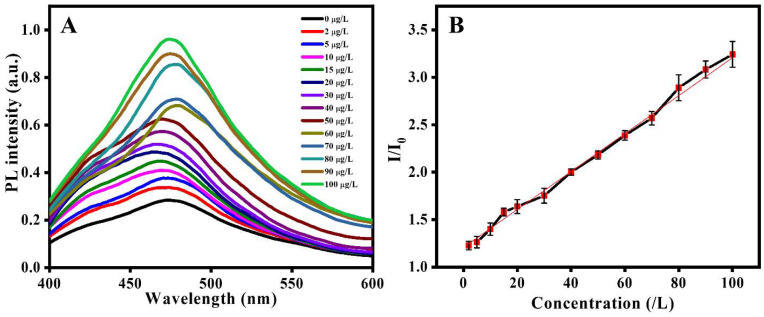
(**A**) Fluorescence response and (**B**) relative fluorescence results for the NPCD/AgNP nanocomposites in the presence of various concentrations of MFH.

The fluorescence spectra of the NPCD/AgNP nanocomposites upon the addition of various concentrations of MFH are shown Figure 8. The intensity of the nanocomposites was steadily recovered at MFH concentrations of 2–100 µg/L. The linear response could be expressed using the equation I/I_o_ = 1.20691 + 0.0199 C_M_, with a correlation coefficient (R^2^) of 0.99308. The sensing experiment was repeated three times, and the data were plotted along with the standard deviations. Moreover, the limit of detection was calculated based on the formula as follows [38]:

LOD = 3σ/k

where σ is the standard deviation of the blank solution and k is the slope of the calibration curve. With σ and k values of 0.0117 and 0.0199, respectively, the LOD was calculated to be 1.76 μg/L. Table 1 shows a comparison of the results obtained using our MFH sensing method and those obtained with other methods reported previously.

### 4.3. Determination of MFH in Human Serum

Practicality and reliability are important factors in evaluating the effectiveness of a sensor. Hence, we attempted MFH detection in human serum using our NPCD/AgNP nanocomposite sensor. Table 2 suggests that the recovery of MFH ranges from 95.7% to 138.8%. The experiment was performed three times and the relative standard deviations (RSDs) were less than 5%. Thus, this is a practical approach for the potential sensing of MFH.

### 4.4. Selectivity

A good sensor must also be highly selective for analyte detection. We investigated the specificity of the NPCD/AgNP nanocomposite material for MFH detection. MFH sensing is often performed in human serum, human urine, and pharmaceutical samples. Hence, to confirm the anti-interference ability of the nanocomposite sensor, the fluorescence responses of some amino acids, carbohydrates, and electrolytes were studied.

**Figure 9:**
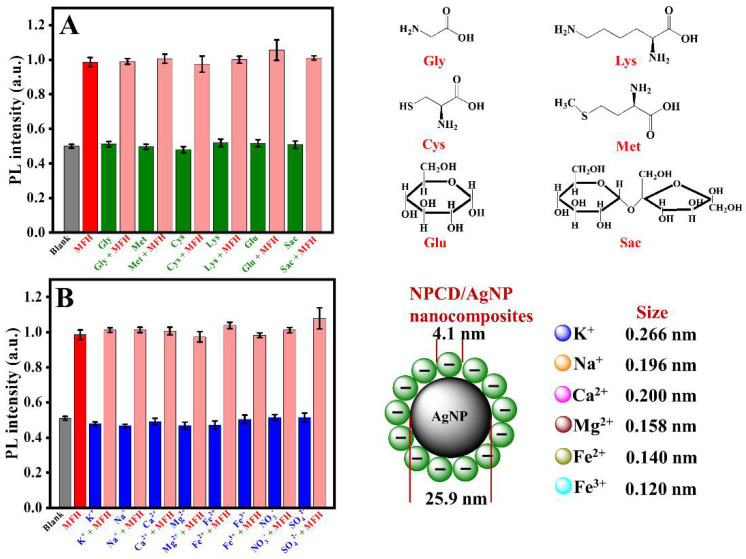
Fluorescence response of the NPCD/AgNP nanocomposites upon the addition of 50 µg/L of different substances (**A**), and ions (**B**).

As shown in Figure 9, in the absence of MFH, the introduction of other substances and ions into the NPCD/AgNP nanocomposite solution could not recover the fluorescence. However, the fluorescence was “turned on” when MFH was added in the presence of other compounds. In other words, the other compounds did not interfere in MFH sensing.

As we stated, the sensing mechanism is based on electrostatic interactions between the negatively charged nanocomposites and the positively charged MFH molecules. Therefore, MFH molecules must be in a protonated state. According to the previous research, in aqueous solution and at physiological pH values ranging from 6.9 to 7.4, metformin (MET) remains in the mono-protonated state. There are two possible protonation sites and these two protonated forms can tautomerise to produce another protonated state. MFH is a protonated form of MET [46]. Therefore, to ensure an electrostatic interaction with the negatively charged NPCD/AgNP nanocomposites, first amino-rich acids such as lysine or other amino acids and compounds need to be in the protonated state in the solution at a pH value of 7.4. However, the protonation possibilities of the substances differ. Moreover, steric hindrance is one of factors that is often exploited to control selectivity because it affects the interactions between molecules. The differences in structure between the compounds result in the various steric hindrances. Therefore, there is a difference in the interaction possibilities of MFH and other compounds to the negatively charged NPCD/AgNP nanocomposites; thus, the other amino acids and compounds could not act as MFH to “turn on” the fluorescence of the nanocomposites.

Although electrostatic interactions with negatively charged NPCD/AgNP nanocomposites are expected upon the addition of certain representative cations such as Na^+^, K^+^, Ca^2+^, Mg^2+^, and Fe^2+^, there was no fluorescence recovery. This is because the AgNPs and the surrounding NPCDs in the NPCD/AgNP nanocomposites have average sizes of 25.9 and 4.1 nm, respectively, while the cations listed in Figure 9 are smaller in size. Therefore, although the cations are involved in electrostatic interactions with NPCDs, the interactions are not strong enough to remove the NPCDs away from the vicinity of the AgNP surface; thus, the IFE between NPCDs and AgNPs in the nanocomposites cannot be effected.

## 5. Conclusions

In conclusion, the prepared NPCDs exhibited excellent performance as a reducing agent and a fluorescence source. Hence, a NPCD/AgNP nanocomposite material was successfully synthesized and effectively utilized as an ideal IFE pair to fabricate a “turn on” fluorescence sensor for MFH detection. The fluorescence of the NPCD/AgNP nanocomposites was “turned on” when MFH was added to the solution, and the intensity of the nanocomposites increased steadily with increasing MFH concentration. A linear response with an R^2^ value of 0.99308 and LOD of 1.76 µg/L was obtained in the MFH concentration range of 2–100 µg/L. The design and operation of the fabricated MFH sensor are simple and do not require expensive instruments. Moreover, compared to other reported methods, our method is more sensitive, with a lower linear range and LOD value. Therefore, the simplicity and sensitivity of this sensor render it potentially viable for MFH sensing and other biomedical sensing applications.

## Figures and Tables

**Table 1:** Results of MFH detection using our method and previously reported methods.

Method	Linear Range	LOD	Reference
RPHPLC HPLC-UV HPLC Voltametric	0.5–4 µg/mL 10 –30 µg/mL 5–150 µg/mL 0.165–662 µg/mL	0.89 µg/mL 0.08 µg/mL	[39] [40] [41] [42]
Voltametric Elecrochemical-FIA CE	0.132–0.993 µg/mL 0.17–16600 µg/mL 0.25–3.5 µg/mL	0.011 µg/mL 0.13 µg/mL 0.1 µg/mL	[12] [43] [44]
LC-MS/MS Fluorescence	2.5×10^−4^–0.01 µg/mL 2×10^−3^–0.1 µg/mL	2.5×10^−4^µg/mL 1.76×10^−3^µg/mL	[45] Our method

**Table 2:** Detection of MFH in human serum.

**Sample**	**Added (µg/L)**	**Founded (µg/L)**	**Recovery (%)**	**RSD** **(*n* = 3)**	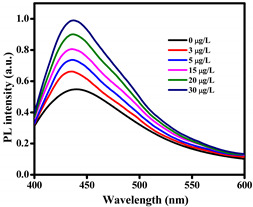
1	3	3.5	118.3	7.3	
2	5	6.9	138.8	4.4	
3	15	14.4	95.7	1.9	
4	20	23.3	116.6	4.8	
5	30	30.9	102.9	3.5	

## Data Availability

Not applicable.
